# Broadband Entrainment of Striatal Low-Threshold Spike Interneurons

**DOI:** 10.3389/fncir.2020.00036

**Published:** 2020-06-12

**Authors:** Juan C. Morales, Matthew H. Higgs, Soomin C. Song, Charles J. Wilson

**Affiliations:** ^1^Department of Biology, University of Texas at San Antonio, San Antonio, TX, United States; ^2^Skirball Institute of Biomolecular Medicine and Neurosciences Institute, New York University School of Medicine, New York, NY, United States

**Keywords:** oscillations, phase-resetting, basal ganglia, resonance, interneuron

## Abstract

Striatal interneurons and spiny projection (SP) neurons are differentially tuned to spectral components of their input. Previous studies showed that spike responses of somatostatin/NPY-expressing low threshold spike (LTS) interneurons have broad frequency tuning, setting these cells apart from other striatal GABAergic interneurons and SP neurons. We investigated the mechanism of LTS interneuron spiking resonance and its relationship to non-spiking membrane impedance resonance, finding that abolition of impedance resonance did not alter spiking resonance. Because LTS interneurons are pacemakers whose rhythmic firing is perturbed by synaptic input, we tested the hypothesis that their spiking resonance arises from their phase resetting properties. Phase resetting curves (PRCs) were measured in LTS interneurons and SP neurons and used to make phase-oscillator models of both cell types. The models reproduced the broad tuning of LTS interneurons, and the differences from SP neurons. The spectral components of the PRC predicted each cell’s sensitivity to corresponding input frequencies. LTS interneuron PRCs contain larger high-frequency components than SP neuron PRCs, providing enhanced responses to input frequencies above the cells’ average firing rates. Thus, LTS cells can be entrained by input oscillations to which SP neurons are less responsive. These findings suggest that feedforward inhibition by LTS interneurons may regulate SP neurons’ entrainment by oscillatory afferents.

## Introduction

Oscillatory synaptic inputs can entrain spiking in neurons, but in each cell type some input frequencies are more effective than others. Frequency-selectivity is one way in which cell types within a local circuit can specialize in the processing of complex input patterns. This phenomenon, called spiking resonance, applies not only to oscillatory signals that are large and easily distinguished from other components of the input, but also to the multiple frequency components embedded in a broadband synaptic input (e.g., Wilson et al., 2018). All signals can be decomposed into a set of constituent sinusoids, so frequency-selectivity affects the responses of neurons to all input patterns. Spiking resonance can be measured as entrainment to experimentally applied sinusoidal input, or as selective entrainment to frequency components in a noisy input.

Cells vary in spiking resonance according to cell type, some showing very sharp frequency selectivity and others broad sensitivity. Some have a relatively fixed frequency sensitivity and in others the frequency sensitivity can be altered flexibly (Beatty et al., 2015). These differences are frequency signatures of cell types and determine the rate and timing of their responses to specific patterns of synaptic input. In a circuit like the mammalian striatum, in which there are diverse cell types sharing synaptic input from the same set of afferents, cells of different types may respond preferentially to different frequency components in the shared input. In the striatum, spiny projection (SP) neurons are tunable, being very selective for input frequencies matching their individual firing rates (Wilson, 2017). In contrast, somatostatin/neuropeptide-Y containing low-threshold spiking (LTS) interneurons are sensitive to a broad range of input frequencies that depends less the cell’s firing rate (Beatty et al., 2015). LTS interneurons provide feedforward inhibition to SP neurons, and their broad frequency sensitivity might oppose SP cell entrainment over a range of input frequencies, altering their effective frequency tuning.

Two different cellular mechanisms can produce spiking resonance: membrane impedance resonance and spike-induced resonance. Membrane impedance resonance is a property of a cell’s constituent subthreshold voltage-dependent ion channels, increasing the subthreshold voltage response to periodic input currents at some frequencies. Membrane impedance resonance is independent of spiking (e.g., Hutcheon and Yarom, 2000) and is best measured in its absence. In cells that have a stable resting membrane potential it can be measured in current clamp recordings by applying sinusoidal currents at various frequencies and determining the amplitude of voltage changes as a function of frequency. In cells that fire spontaneously this approach is not available, but spiking can be controlled in voltage clamp, and impedance is measured as the inverse of current generated by the membrane in response to sinusoids of applied voltage (e.g., Beatty et al., 2015). The second mechanism of spiking resonance arises from ion channels triggered by action potentials; this mechanism is not operational when the cell is silent. Spike-triggered currents vary in sign and in time course, producing a sequence of changes in sensitivity to inputs following each action potential (e.g., Yarom et al., 1985; Higashi et al., 1993). Periodic synaptic inputs that are properly aligned with the sequence of spike-induced intrinsic currents gain an advantage in entraining firing. Theoretical and simulation studies have explored the relative strength of these two mechanisms of spiking resonance and possible interactions between them (e.g., Richardson et al., 2003; Rotstein, 2017) and suggest that they might interact to produce the spiking resonance phenomena observed in neurons. Of course, subthreshold-activated ion channels contribute to the trajectory of the neuron between action potentials, and so membrane impedance resonance and spike-dependent mechanisms are not easily separable in experiments when cells are firing.

Striatal SP neurons have no membrane impedance resonance, so their spiking resonance arises solely from spike-dependent mechanisms. That explains why their frequency sensitivity follows their own firing rate (Beatty et al., 2015; Wilson, 2017). Striatal LTS interneurons do have a membrane impedance resonance, and so it is possible that their spiking resonance depends on an interaction between spike-induced and spike-independent resonance mechanisms. The mechanism of membrane impedance resonance in striatal LTS interneurons is known (Song et al., 2016), and it depends on calcium influx through Cav2 channels. This offers the opportunity to remove membrane impedance resonance by blocking those channels and determine the contribution of this mechanism to spiking resonance. In the work reported here we show that membrane impedance resonance has no measurable contribution to the spiking resonance of striatal LTS interneurons or their entrainment by periodic current waveforms. The characteristic features of the LTS cell’s spiking resonance can all be traced to spike-induced changes in input-sensitivity that can be measured using phase resetting.

## Materials and Methods

All experimental procedures were in accordance with the National Institutes of Health *Guidelines for the Care and Use of Laboratory Animals* and were approved by the Institutional Animal Care and Use Committee of the University of Texas at San Antonio.

### Brain Slice Preparation

Transgenic mice (B6.FVB-Tg(Npy-hrGFP)1Lowl/J) expressing the green fluorescent protein (GFP) reporter under the control of the neuropeptide-Y (NPY) promoter (Jackson Laboratories, stock no. 006417) were deeply anesthetized with isoflurane and perfused intracardially with a sodium-free, chilled solution containing (in mM) 2.5 KCl, 1.25 NaH_2_PO_4_, 0.5 CaCl_2_, 10 MgSO_4_, 10 D-glucose, 26 NaHCO_3_, and 202 sucrose at pH 7.4. Immediately after perfusion, mice were decapitated and their brains extracted for the slicing procedure. 300 μm parasagittal slices containing the striatum were cut in the chilled solution used for perfusion (described above) using a vibrating slicer. Slices were collected in bubbled (95% O_2_–5% CO_2_) artificial cerebrospinal fluid (ACSF), which consisted of (in mM) 126 NaCl, 2.5 KCl, 1.25 NaH_2_PO_4_, 2 CaCl_2_, 2 MgSO_4_, and kept at room temperature for at least 1 h before transfer to the recording chamber for perforated patch recordings.

### Electrophysiological Recordings

Slices were bathed continuously with oxygenated ACSF at a rate of 2–3 mL/min and kept at 35°C. We used the perforated patch method to reduce cell dialysis and prevent rundown of spontaneous firing. Micropipettes with 4–8 MΩ tip resistances were filled with a solution containing 140.5 mM KMeSO_4_, 7.5 mM NaCl, 10 mM HEPES, and 0.5 μg/ml gramicidin. Gramicidin was mixed in DMSO (0.5 mg/ml) and diluted (1:1000) in filtered electrode solution. Synaptic transmission was not blocked, as injected currents were applied to single neurons. Blockade of GABAergic transmission was considered unnecessary, as other spiny neurons were mostly inactive in our slices, and LTS interneurons have been reported not to be strongly interconnected (Gittis et al., 2010).

Striatal LTS cells and spiny neurons were targeted with an Olympus BX50WI upright microscope using the water-immersion 40x objective. Data were acquired using a Multiclamp 700B amplifier (Molecular Devices, Union City, CA, United States), filtered at 10 kHz, digitized at 20 kHz by a HEKA ITC-18 analog-to-digital converter, and collected using Igor Pro (WaveMetrics, Lake Oswego, OR, United States). All data analysis was done offline using *Mathematica* (Wolfram Research, Champaign, IL, United States).

### Measuring Entrainment of Spiking

To measure entrainment, cells of both types were stimulated with a sequence of sine wave currents varying in frequency with amplitude of 20 pA. The frequency of the sine wave was varied from 1 Hz to 25 Hz, in 1 Hz steps. Spike times were detected offline. For each sine wave frequency (*f*), each spike time (*t_*i*_)* was converted to phase (0≤θ < 1) relative to the period of the sine wave using the Mod operator:

$$\theta_{f,i}=M\,o\,d\,\left(f\cdot t_{i},1\right),$$

The phase probability distribution for each frequency was then calculated using 100 bins to represent phases from 0 to 1. We then calculated the entropy of each phase distribution, normalized by the expected entropy of a sample of the same size drawn from the uniform phase distribution, *E(H_*u*_)*,

$$H=\frac{-\sum_{i=1}^{N}p_{i}\,l\,o\,g_{2}\,\left(p_{i}\right)}{E\,\left(H_{u}\right)}.$$

This entropy measure removes any bias from differences in the number of spikes collected in each sample. Perfect entrainment (i.e., all phases falling within one of the 100 bins of the phase distribution) would produce an entropy value of zero. At the other extreme, sampling from a uniform distribution of spike phases would produce an expected entropy value of one.

### Measuring Spiking Resonance

**FIGURE 1:**
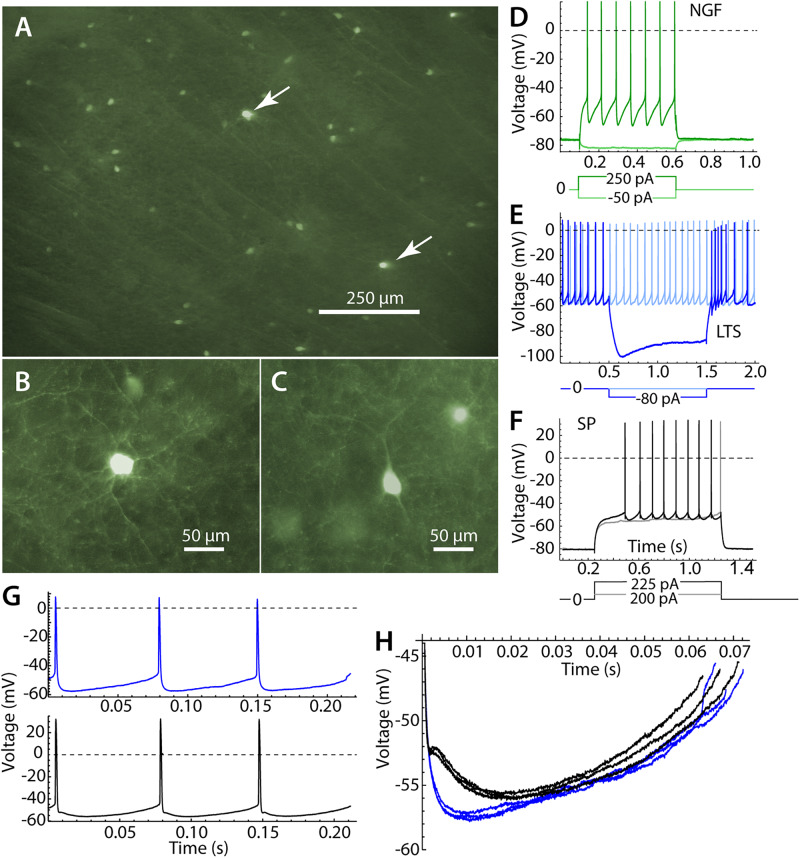
Identification of striatal neurons. **(A)** Panoramic view of the striatum in parasagittal section. Most labeled cells are LTS interneurons, but there are a few neurogliaform (NGF) cells, which are brighter (arrows). **(B)** A NGF cell at higher magnification, characterized by many primary dendrites that branch frequently. **(C)** An LTS interneuron, with fewer and straighter dendrites. **(D)** Responses of an NGF cell to hyperpolarizing and depolarizing current pulses. Note the stable hyperpolarized membrane potential in the absence of current. **(E)** An LTS interneuron, with continuous autonomous firing in the absence of current, and a prominent rebound burst at the offset of a hyperpolarizing current pulse. **(F)** A SP neuron, with characteristic long delay to first spike in response to depolarizing currents. **(G)** Repetitive firing of an LTS interneuron in the absence of injected current (blue) and an SP neuron, firing repetitively (black) when depolarized to approximately match the firing rate of the LTS interneuron. **(H)** The interspike membrane potential trajectory of the same LTS interneuron (blue) and SP neuron (black) shown in **(G)**. SP neurons show separate fast and medium afterhyperpolarizations, whereas LTS interneurons have a single afterhyperpolarization.

To measure spiking resonance, LTS cells were stimulated for 160 s with a barrage of contiguous brief (0.5 ms) current pulses, whose amplitudes were drawn from a Gaussian distribution (mean = 0 pA and standard deviation = 40 pA). For some experiments we substituted a 1000/s Poisson-triggered barrage of artificial synaptic currents with 20 pA amplitudes and 2.5 ms time constants, as described in Beatty et al. (2015). These two stimulus waveforms differ only at high frequencies, and they produced the same results. In either case, each injected current waveform was Fourier transformed in *Mathematica* and filtered in the frequency domain by multiplying the frequency-domain waveform by Tukey filters (α = 0.25) with bandwidths of 10 Hz, centered at every 5 Hz from 5 to 110 Hz. This resulted in 22 filtered signals which were then transformed back into the time domain. Spikes were assigned phases on these waveforms by finding the zero-crossings of the signal, finding the time of each spike from the left positive-slope zero-crossing, and dividing by the length of that cycle (see Figure 1). The vector strength was then calculated for phases corresponding to each of the 22 filtered signals by:

$$v\,s\,\left(f\right)=\left|\frac{1}{N}\,\sum_{j=1}\left(c\,o\,s\,\left(2\,\pi \,\phi_{j}\right)+i\,s\,i\,n\,\left(2\,\pi \,\phi_{j}\right)\right)\right|,$$

which is the magnitude of the normalized vector sum. This resulted in a value ranging from zero to one, with one indicating perfect phase locking and zero meaning no locking.

### Measuring Phase Resetting Curves

The method used for experimentally measuring phase resetting curves (PRCs) has been described previously (Netoff et al., 2011; Wilson et al., 2014). A sequence of contiguous 0.5 ms current pulses whose amplitudes were independently drawn from a Gaussian distribution (mean = 0 pA and standard deviation = 40 pA) was injected into repetitively firing LTS or spiny cells through the recording electrode for 160 s. This stimulus was the same as one of the two used to measure spiking resonance, so it was possible to measure spiking resonance and PRCs from the same cell. Phase within each interspike interval was estimated by interpolation (i.e., the estimated phase ramped linearly from 0 to 1 between spikes) and was discretized into 50 bins. The charge delivered during each bin was calculated by integration of the noise waveform for each bin in each interspike interval during the noise stimulus. Because the charge delivered in each phase bin was different on each ISI, it was possible to calculate linear regressions between charge and ISI length by multiple linear regression. The slopes of these regressions, normalized by the average ISI, produced the 50 values that make up the PRC. The standard errors for each of the estimates of Z were also calculated and are shown as error bars around the PRC values.

We measured the duration of individual inter-spike intervals (*ISI*_*n*_), the average inter-spike interval $\left(\bar{I\,S\,I}\right)$, and the charge (*Q*) delivered per phase bin (in all cases *m* = 50), to solve for the PRC values (*Z*_*m*_) at m phase bins using multiple regression, minimizing the mean squared value of the residual ξ_n_:

$$\frac{I\,S\,I_{n}-\bar{I\,S\,I}}{\bar{I\,S\,I}}=\sum_{i=1}^{m}Q_{n,i}\,Z_{i}+\xi_{n}$$

Calculation of the PRC was done in *Mathematica* using the built-in function, *LinearModelFit.*

### Phase Modeling

We implemented a version of the phase model containing intrinsic noise and an external stimulus (either pulsed noise current or sinusoidal current). The phase model describes the temporal evolution of phase and is governed by the differential equation:

$$\frac{d\,\varphi }{d\,t}=F_{c\,e\,l\,l}+\left[I_{\text{stim}}\,\left(t\right)+\eta \,\left(t\right)\right]\,Z\,\left(\varphi \right)$$

$$\varphi \,\left(0\right)=\varphi_{0}$$

where φ is the cell’s intrinsic phase, *F*_*cell*_ is the cell’s measured unperturbed firing rate, *I*_stim_ the stimulus current delivered through the recording electrode, η is the cell’s estimated intrinsic noise, and *Z* is the PRC measured using multiple regression (outlined above). Phase is only defined from zero to one, with one indicating a spike, so when phi reached one, it was reset back to zero.

## Results

### Cell Identification

Striatal LTS interneurons were identified by fluorescence microscopy in brain slices from NPY-GFP mice at the time of recording. We were careful not to include recordings from neurogliaform (NGF) cells, which are also fluorescent in the mouse line used in our study (Ibáñez-Sandoval et al., 2011). NGF cells were identifiable by their brighter fluorescence and their characteristic morphological and physiological properties (Ibáñez-Sandoval et al., 2011). Examples of the morphology and responses of LTS and NGF cells are shown in Figure 1. All LTS cells used in the sample had the morphology and responses to current steps characteristic of striatal LTS cells (Kawaguchi, 1993; Tepper et al., 2010; Beatty et al., 2012). These include sustained autonomous rhythmic firing and rebound bursts following hyperpolarizing current steps, neither of which were seen in NGF cells. LTS cells in the persistent depolarized state (Song et al., 2016) were not included in our sample.

Recordings of SP neurons were taken from a previous dataset (Wilson, 2017). Half of the SP cells were identified as either direct or indirect pathway neurons in mice expressing tdTomato or GFP under control of the D1 or D2 receptor promoter, respectively. The remainder were SP neurons identified by their characteristic physiological properties: low input resistance, hyperpolarized resting potential, fast inward rectification, and late first spike in response to a near-threshold depolarizing pulse. Entrainment of SP cells by sine wave currents was reported in the previous paper (Wilson, 2017) and is included here only for comparison to LTS cells. The spiking resonance results reported here have not been presented previously, but a similar measurement in a different sample of SP cells was reported in Beatty et al. (2015). D1R and D2R neurons did not differ in spiking resonance and so results from all SP cells of both types were pooled.

Low threshold spike interneurons are autonomously active in slices and measurements of sinusoidal entrainment and spiking resonance were made at their spontaneous firing rates. Spiking resonance was measured in 18 LTS interneurons with firing rates ranging from 6.9 to 23.8 spikes/s (mean = 15.4, sd = 5.7). Because striatal SP neurons are silent in slices, we used constant current to evoke repetitive firing in these cells. The current was adjusted to achieve firing rates in the same range as those of LTS interneurons. Firing rates in 18 SP cells ranged from 11.1 to 19.8 spikes/s, mean = 14.0, sd = 2.6). When firing at comparable rates, the interspike membrane potential trajectories of LTS interneurons and SP neurons traversed a similar range, but LTS interneurons had deeper and faster spike afterhyperpolarizations (Figures 1G,H).

### Spiking Resonance

**FIGURE 2:**
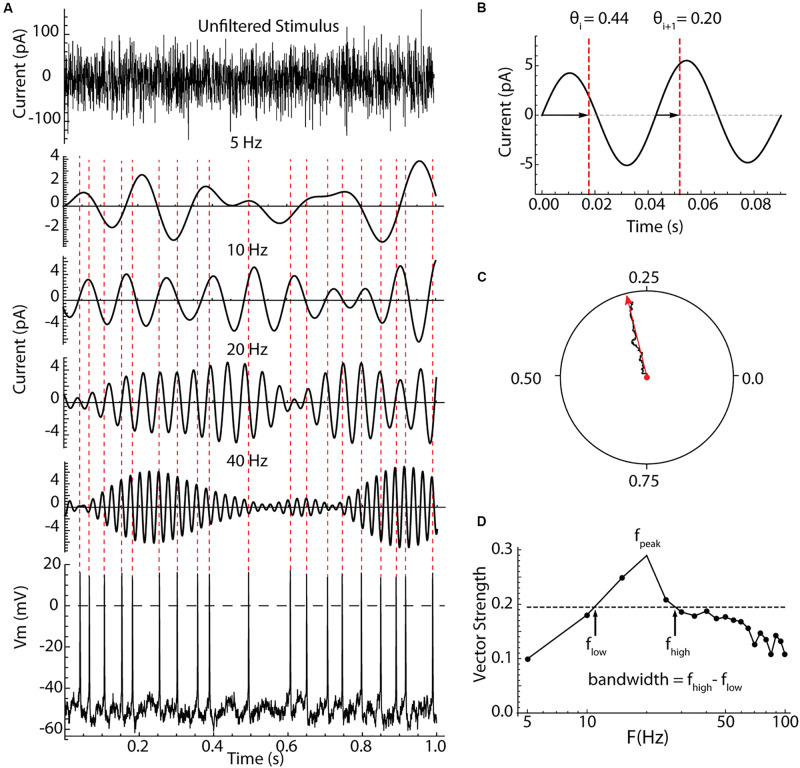
Measurement of spiking resonance. **(A)** An example 1 s of the injected current (top), bandpass filtered components of the same current centered on different frequencies, and the resulting voltage waveform and perturbed spiking in a striatal LTS interneuron. The spike times are indicated with dotted lines crossing the filtered current waveforms. **(B)** Measurement of spike phases on the filtered waveform. Phase was measured between positive-slope zero crossings. **(C)** The summation of phase measurements from many action potentials as a vector sum. The resultant vector in red, points in the direction of the average phase, and its length is the vector strength. **(D)** A spectrum of vector strength measurements from an example LTS interneuron taken for a range of bandpass center frequencies. The frequency producing the peak resonance, *f*_peak_, and the half-width of the spectrum (bandwidth) were measured as shown using the half-height taken between the maximum and minimum vector strengths.

The measurement of spiking resonance is illustrated in Figure 2. Cells were recorded in the perforated patch configuration, and electrode capacitance was compensated to ensure accurate delivery of charge to the cell membrane. In some cells, the stimulus was a Poisson-timed 1000/s barrage of synaptic-like currents averaging 20 pA in amplitude, with decay time constants of 2.5 ms (see Figure 4). In the rest of the cells (as shown in Figure 2), contiguous pulses of current 0.5 ms in duration with amplitudes drawn from a Gaussian distribution with a mean of zero and a standard deviation of 40 pA were injected through the somatic recording electrode. These two stimulus waveforms both have constant spectra over most of the frequency range of interest, and they yielded similar spiking resonance measurements. An example showing the appearance of the current waveform, and its effect on the firing pattern of a repetitively firing cell is shown in Figure 2A. To analyze spiking resonance, the current waveform was digitally filtered into 22 frequency bands, each 10 Hz wide, centered on frequencies from 5 to 110 Hz in 5 Hz intervals. These were chosen to cover the range of membrane and spiking resonances previously reported for LTS interneurons, with resolution sufficient to characterize the broad resonance peaks of those cells (Beatty et al., 2015; Song et al., 2016). Examples of the filtered current waveforms for selected frequency bands are shown in Figure 2A. Like the original stimulus waveform, each filtered waveform had a mean of zero. The stimulus altered the timing of spikes, with no consistent effect on average firing rate. The spike times were converted to phases on each of the filtered waveforms. Spike phase was defined as the proportion of the cycle between positive-slope zero crossings of the filtered waveform bracketing the spike, as shown in Figure 2B. The phases of all spikes were vector-summed to make a resultant vector, as shown in Figure 2C. The lengths of the resultant vectors (vector strengths) potentially range from zero (meaning phases are uniformly distributed with respect to the filtered stimulus waveform) to one (if every spike is at exactly the same phase on the filtered stimulus). The vector strengths for all the filtered waveforms were used to make a spectrum of spiking resonance, as shown in Figure 2D. The peak frequency and half-width (bandwidth Figure 2D) were measured from the spiking resonance spectrum.

**FIGURE 3:**
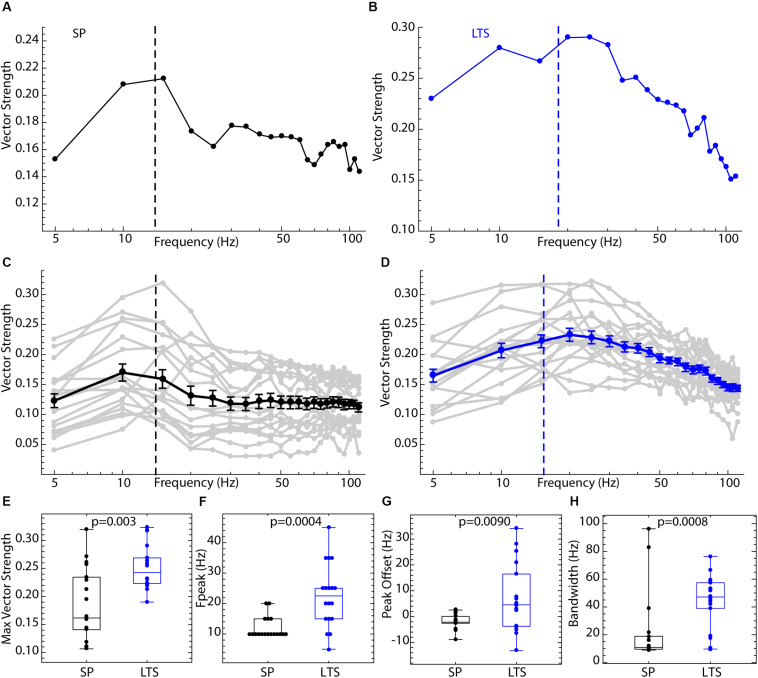
Comparison of spiking resonance in SP neurons and LTS interneurons. **(A)** An example spiking resonance spectrum from an SP neuron. The cell’s firing rate is indicated by the dotted line. **(B)** An example LTS interneuron shown the same way. **(C)** Superimposed spiking resonance spectra (gray) and the average spiking resonance (black) for 18 SP neurons. **(D)** Superimposed individual spectra (gray) and average spectrum (blue) for 18 LTS interneurons. Error bars are standard errors of the mean at each frequency. The dotted lines indicate the average firing rates for the two groups of cells. **(E)** LTS interneurons showed a higher vector strength overall than SP neurons. **(F)** The frequency at the peak resonance was higher in LTS interneurons. **(G)** The difference between the firing rate and peak frequency for each cell was greater in LTS interneurons. **(H)** The bandwidth of resonance (measured as shown in Figure 2) was greater in LTS interneurons.

Spiking resonance spectra for an example LTS interneuron and SP neuron, and the average spectra for both cell types, are shown in Figure 3. The spectra for the two cell types differed in overall amplitude, the peak frequency, and the bandwidth, as defined in Figure 2. Example spectra are shown in Figures 3A,B. Spiking resonance spectra for all cells in each group are superimposed with the group means in Figures 3C,D. On average, LTS interneurons showed larger vector strengths at all frequencies. That is, spike timing was more coherent with all frequency components of the stimulus waveform. The maximum value of each spiking resonance spectrum for both cell types is shown in Figure 3E. This difference between cell types was statistically significant (Mann–Whitney *U*, *p* = 0.0005). We have previously shown (Wilson, 2017) that D2R-expressing SP neurons have greater sensitivity to periodic inputs than D1R-expressing ones, and this accounts for some of the variation among SP neurons in overall vector strength. The peak of the spiking resonance spectrum occurred at a lower frequency for the SP neurons (Figure 3F, Mann–Whitney *U*, *p* = 0.0004). In SP neurons, the peak frequency for spiking resonance varies with firing rate (Beatty et al., 2015). In LTS interneurons firing at the same average rate as the SP neurons, the peak frequency was at a higher frequency, usually higher than the cell’s average rate (Figure 3D). Because the peak frequency of resonance in both cell types depended to some degree on firing rate, we measured the difference between the firing rate and the peak in the spiking resonance spectrum for each cell. This measure was significantly different between the two cell types (Figure 3G, Mann–Whitney *U*, *p* = 0.009). The resonance bandwidth (measured as shown in Figure 2D) was broader for LTS interneurons than SP neurons, as also evident in Figure 3H (Mann–Whitney *U*, *p* = 0.0008).

**FIGURE 4:**
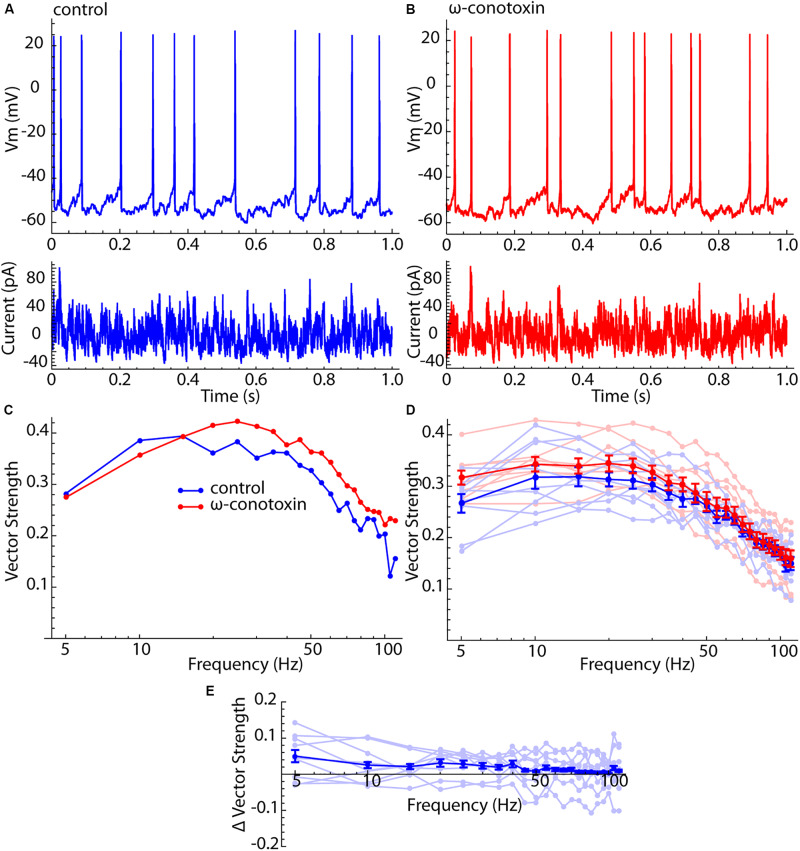
Lack of effect of Cav2 channel blockade on spiking resonance in LTS interneurons. **(A)** Current waveform, membrane potential, and firing pattern for an example LTS interneuron. **(B)** The same example neuron after treatment with 1 μM ω-conotoxin GVIA. **(C)** Spiking resonance spectra for an example LTS interneuron before (blue) and after (red) conotoxin treatment. **(D)** Superimposed spectra for all cells before (light blue) and after (light red) conotoxin treatment, and the mean spectra. Error bars are standard errors of the mean. **(E)** Difference spectra (conotoxin – control) for each cell before and after conotoxin treatment (light blue) and the mean difference spectrum. There was no significant frequency trend for the difference spectra (*F* = 0.68, df = 8,21, *p* = 0.85).

### No Contribution From Membrane Impedance Resonance

It was proposed previously that the enhanced sensitivity of LTS interneurons to input frequency components higher than their firing rates might represent a contribution from their membrane impedance resonance (Beatty et al., 2015). Unlike SP neurons, LTS interneurons have a substantial membrane resonance, which is generated by a calcium-dependent chloride current that is active only when the cell is depolarized (Song et al., 2016). It was previously shown that the membrane impedance resonance is abolished by blockade of Cav2 channels by 1 μM ω-conotoxin GVIA (Song et al., 2016). To determine whether the peculiar features of spiking resonance in LTS interneurons arose from their impedance resonance, we measured spiking resonance in nine LTS interneurons before and after application of 1 μM ω-conotoxin GVIA. For this experiment, spiking resonance was measured using a barrage of artificial synaptic currents like those used in Beatty et al. (2015) (see the section “Materials and Methods”). Examples of this current waveform, and its effects on membrane potential and spiking in control ACSF and in conotoxin are shown in Figures 4A,B. The corresponding spiking resonance spectra for an example cell are shown in Figure 4C. All spectra are superimposed in Figure 4D, along with the average spectra for both conditions. Because this was a paired comparison, the appropriate test for an effect of conotoxin is the difference spectrum for each cell. These are shown in Figure 4E. There was no statistically significant frequency trend in the difference curve (ANOVA, *F* = 0.68, df = 8,21, *p* = 0.85). Blockade of Cav2 channels with conotoxin also had no statistically significant effect on the overall sensitivity (signed rank test, *p* = 0.19), the position of the peak in the spiking resonance curve (signed rank test, *p* = 0.86), or the resonance bandwidth (signed rank test, *p* = 0.95). It also had no effect on the firing rates of LTS cells; the average rate for the sample was 12.3 ± 2.7 spikes/s, and 12.3 ± 1.7 for the same cells after conotoxin treatment.

The result shown in Figure 4 fails to support the idea that the difference between the spiking resonance spectra of SP neurons and LTS interneurons might result from the LTS interneuron’s membrane impedance resonance.

### LTS Interneurons Are Entrained Over a Broad Frequency Range

The broad range of spiking resonance in LTS interneurons suggests that they might also be entrained by sinusoidal inputs over a broad range of frequencies. In SP neurons, there is a strong correspondence between spiking resonance measured using broadband stimuli and the entrainment profile to single-frequency sine wave inputs. Like the spiking resonance, entrainment to single sine waves in SP neurons is strong only for stimulus frequencies close to the cell’s unperturbed firing rate (Wilson, 2017). Does the broadband spiking resonance of LTS interneurons mean that they have a broader frequency range of entrainment?

**FIGURE 5:**
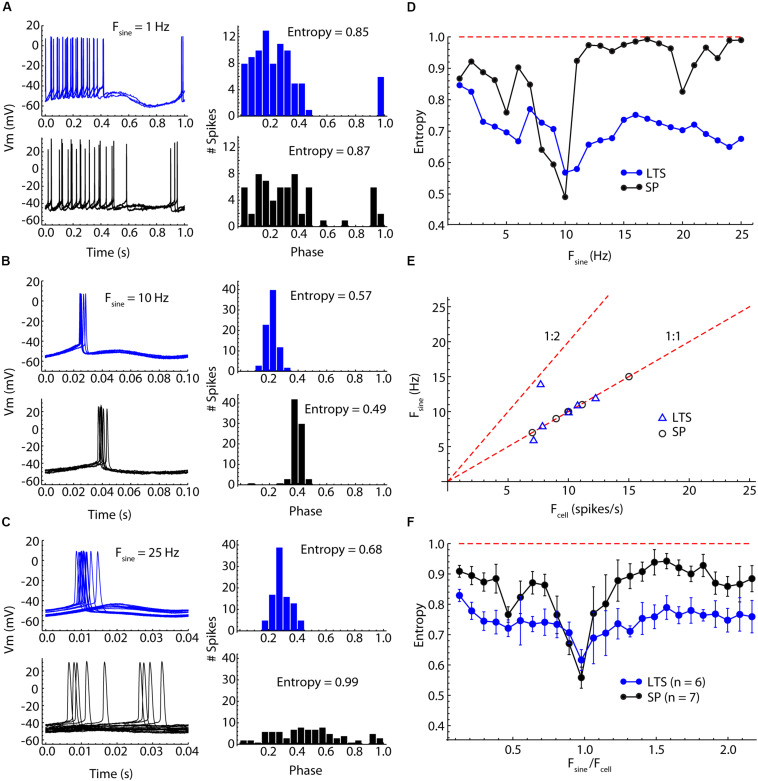
Comparison of SP neuron and LTS interneuron entrainment by injected sinusoidal currents of frequencies between 1 and 25 Hz. **(A)** Low-frequency (1 Hz) sine wave stimulation produced firing rate modulation in both cell types, with neurons firing throughout the positive phase of the stimulus but with little spike time reliability. Example traces for one cycle of the stimulus are shown on the left, and a histogram of spike phases on the stimulus is shown on the right. The peak of the sine wave is phase 0.25, and the trough is phase 0.75. The degree of entrainment is measured as the entropy of the histogram, with low entropies meaning better entrainment. **(B)** Sinusoidal current waveforms at a frequency near the cells’ unperturbed firing rates (about 10 Hz in both cells) evoke 1:1 entrainment of firing at a reliable phase of the stimulus in both cell types. **(C)** At frequencies higher than the cell’s firing rate, 1:1 entrainment failed, but LTS interneurons continued to be more entrained than SP neurons, whose spike times were almost completely unrelated to the stimulus sine wave. **(D)** Entrainment spectra for the example neurons used in **(A–C)**. Entrainment was better in the LTS interneuron at all frequencies except for those close to the cells’ unperturbed firing rates. The difference is especially clear at frequencies near 1.5 times the unperturbed firing rates, at which the SP neurons showed almost no entrainment. The expected entropy for zero entrainment is indicated by the dotted line. **(E)** The sine wave frequency producing peak entrainment (*F*_sine_) was close to the cells’ unperturbed firing rate (*F*_cell_) in both cell types. One exception is an LTS interneuron that had slightly stronger entrainment at twice the unperturbed firing rate. **(F)** Mean entrainment profile for a sample of six LTS interneurons and seven SP neurons. *F*_sine_ is plotted as a proportion of *F*_cell_. Note the absence of any second entrainment peak corresponding to the membrane impedance resonance. Compared to SP neurons, LTS neurons are much less frequency-selective, showing substantial entrainment over the entire frequency range.

We compared the entrainment frequency selectivity of six striatal LTS interneurons to that of seven SP neurons. As in the spiking resonance measurements LTS interneurons were allowed to fire at their natural rates. Repetitive firing in SP neurons was evoked by constant current adjusted to achieve firing rates comparable to those of LTS cells. In both cell types, 20 pA sine wave currents from 1 Hz to 25 Hz at 1 Hz steps were applied for 8 s each. Spike times were converted to phases relative to the stimulus sine wave, and phase histograms were constructed as in the examples shown in Figures 5A–C. To quantify entrainment, we estimated the deviation of the spike phase distribution from the uniform distribution expected in the absence of any entrainment by calculating the entropy of the histograms. Entropy measures depend on the sample size (number of action potentials) and on the number of bins in the histogram, so to correct for these factors we calculated the expected entropy for a uniform phase distribution for each sample and normalized the measured entropy by that value. This normalized entropy varies from 0, meaning all spikes occurred in the same phase bin, to 1, indicating no entrainment at all.

Low-frequency sinusoidal current modulated firing rate in phase with the stimulus in both cell-types (Figure 5A). As reported previously (e.g., Kawaguchi, 1993), LTS cells have a prominent calcium-dependent rebound (low-threshold) spike when released from constant hyperpolarization. The sine waves used here were too weak to remove the low-threshold calcium current inactivation and did not evoke a rebound spike (e.g., Figure 5A). At stimulus frequencies low enough to produce rate modulation, spike timing during the cycle was not reproducible, and the entropy of the phase distribution was only moderately reduced. At higher frequencies, both SP neurons and LTS interneurons fired fewer action potentials per cycle, and these preferentially occurred at specific phases, so the entropy of the phase distribution was reduced further. The precision of spike timing was maximal when the stimulus frequency matched the unperturbed firing rate of the neuron (Figure 5B). Some neurons showed another minimum of entropy at frequencies about twice their unperturbed rate (e.g., the SP neuron in Figure 5D). At frequencies between the cell’s unperturbed rate and twice that rate, the entrainment was minimal, often (in SP neurons) approximating random firing with respect to the sine wave. LTS interneurons showed stronger entrainment than SP neurons at frequencies both lower and higher than the 1:1 locking frequency (Figure 5D). At frequencies for which SP neurons showed almost no entrainment by the stimulus, LTS interneurons were still substantially entrained (Figure 5C). There was no specific second frequency of entrainment for LTS interneurons corresponding to their membrane impedance resonance. In contrast with the spiking resonance spectra, entrainment of both LTS interneurons and SP neurons was maximal at their unperturbed firing rates (Figure 5E). The only exception was one LTS interneuron that showed stronger entrainment to a sine wave at twice its firing rate (1:2 entrainment in Figure 5E). To compare cell types, we rescaled the stimulus frequencies by each cell’s firing rate (Figure 5F). LTS interneurons showed substantial entrainment to all stimulus frequencies, whereas SP neurons were very selective, responding especially weakly to stimulus frequencies above the cell’s own rate (Figure 5C). The overall mean normalized entropy of LTS interneurons was significantly lower than that of SP neurons (Figure 5F, *t* = 2.9346, df = 11, *p* = 0.014).

For the SP neuron, which lacks membrane impedance resonance, frequency selectivity arises directly from the cell’s PRC (Wilson, 2017). If the fundamental mechanism of entrainment in SP neurons and LTS interneurons is the same kind of phase resetting, then the wider frequency range of entrainment to sine waves and the broad spiking resonance curves of LTS cells might be explained by the features of their PRCs.

### Phase Resetting Curves of LTS Interneurons and SP Neurons

**FIGURE 6:**
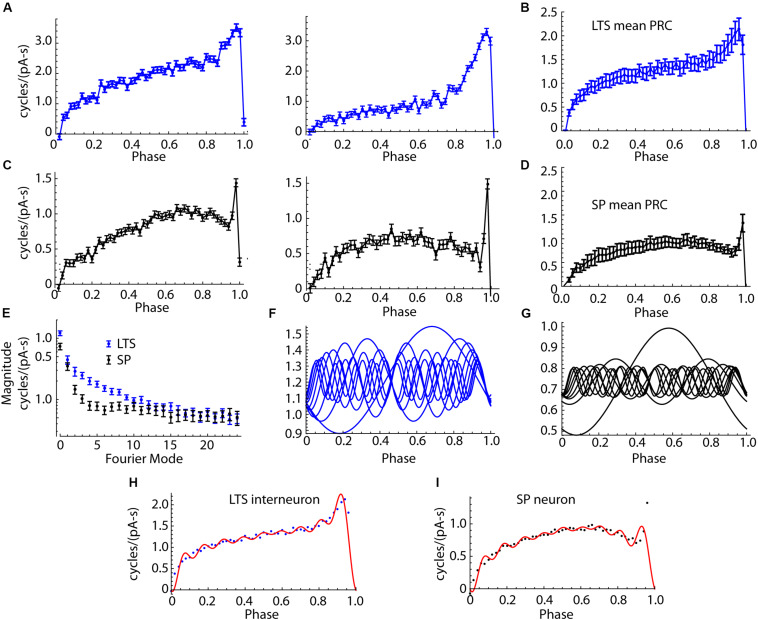
Comparison of PRCs of SP neurons and LTS interneurons. **(A)** Two example LTS interneuron PRCs. **(B)** The mean PRC for the sample of 18 LTS interneurons. Error bars are standard errors of the means. **(C)** Examples PRCs from two SP neurons. **(D)** Mean PRC for the group of SP neurons. The consistent differences in shape include the more pronounced skew in the LTS interneuron PRC and its broader late peak. SP neurons’ PRCs were more symmetrical, except for a very narrow late peak, only one or two bins wide, at the end of the ISI. **(E)** Amplitudes of the Fourier modes of the PRCs of LTS interneurons and SP neurons. 26 modes are shown, calculated from the 50 points in the PRC. Mode 0 is the DC component, which is the average amplitude of the PRC. The differences between cell types are confined to the first 10 modes. **(F)** Modes 0–9 of the average LTS interneuron PRC. **(G)** Modes 0–9 of the average SP neuron PRC. **(H)** Mean LTS interneuron PRC, assembled from the modes shown in **(F)**. Points shown are the original mean PRC. **(I)**. SP neuron average PRC (points) and PRC reassembled from modes shown in **(G)** (red line).

We compared the infinitesimal PRCs of the same 18 LTS interneurons and 18 SP neurons whose spiking resonances are compared in Figure 3. The PRC was estimated from the same pulsed noise stimulus used for the spiking resonance measurement. Example PRCs from both cell types are shown in Figure 6. The PRC is quantified as phase change per picocoulomb (pA-s) of charge injected. The PRC represents the sensitivity of the cell’s spike timing to current perturbations applied at any time between action potentials. The shape of the PRC is determined by the sequence of both spike-triggered and spike-independent currents that are active during the ISI (e.g., Farries and Wilson, 2012). From the PRC, one can predict the cell’s response to any waveform of input current that may be imposed during the ISI, including artificial current or synaptic inputs (Wilson, 2017; Simmons et al., 2018).

SP neurons and LTS interneurons showed substantial differences in PRC size and shape. Examples of LTS interneuron PRCs are shown in Figure 6A, and the average for LTS interneurons is shown in Figure 6B. SP neuron PRCs have a more symmetric shape, peaking at phases near 0.75, and decreasing thereafter, with an extremely brief peak at the end, just before the next action potential. Examples are shown in Figure 6C, and the average PRC for SP neurons is shown in Figure 6D. On average PRC integrals of LTS interneurons were larger than those of SP neurons (0.74 cycles/pA-s for SP neurons and 1.210 for LTS neurons, Mann–Whitney *U*, *p* = 0.00084).

In repetitively firing neurons, sinusoidal inputs alter spike timing by interacting with a corresponding (same frequency) mode in the cells’ PRCs (Goldberg et al., 2013). To understand how PRC shape determines the response to sinusoidal current inputs, we decomposed each neuron’s PRC into sinusoidal components using the discrete Fourier transform. Because the PRC was composed of 50 points, this produced one DC component and 25 sinusoidal components whose sum could reproduce the original PRC. The components (modes) are calculated relative to each cell’s mean ISI, so that the frequency of mode 1 is the cell’s mean firing rate, mode 2 is twice that rate, etc. The amplitudes and phases of the modes determines the size and shape of the PRC. The PRC mode amplitudes of LTS interneurons and SP neurons are compared in Figure 6E. The amplitudes for LTS interneurons were higher than those of SP neurons overall [mixed-design ANOVA, *F*(cell type) = 13.9, df = 1,34, *p* = 0.0007]. The difference was large for mode 0, smallest for mode 1, and large again for modes 2–7. Beyond mode 10, there was little difference between cell types. The first 10 components of the average PRCs from the LTS interneurons and SP neuron are shown in Figures 6F,G, respectively. Reconstruction of the average PRCs of each cell type from their first 10 modes is compared to the original average PRCs in Figures 6H,I. The first 10 modes are sufficient to account for the major features of the PRCs and the differences between cell types. The most notable exception is the extremely narrow peak near phase 1 in the SP neuron.

### Constructing the Phase Models

**FIGURE 7:**
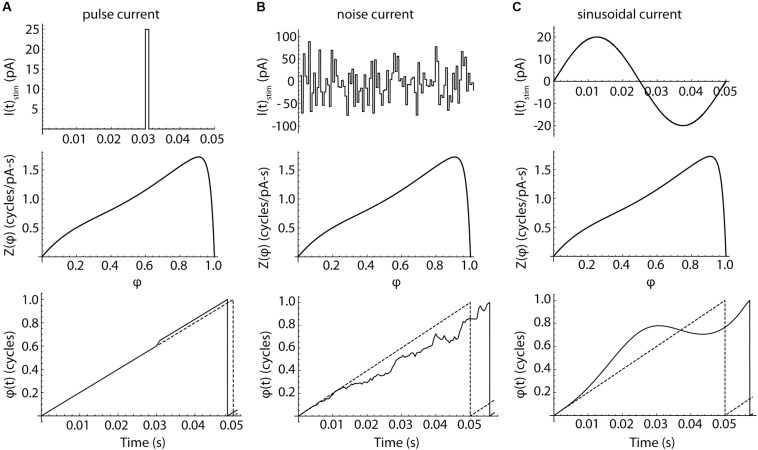
The responses of phase neurons. **(A)** Response to a brief current pulse. Charge delivered by a current pulse (top) causes a phase shift in a noise-free neuron, whose phase otherwise advances at a constant rate *F*_cell_. The cell fires when its phase reaches a value of 1. The depolarizing pulse produces a change in spike time. The size of the phase shift and the change in the spike time are determined by the value of the phase resetting curve at the time of the stimulus. The evolution of phase over time in the absence of any perturbation is indicated by the dotted line. **(B)** Pulsed noise, like that used to measure spiking resonance, produces a sequence of phase shifts. Each pulse arrives at a phase determined by the entire sequence of preceding pulses. Pulses arriving at phases near the peak of the PRC are more influential than others, but the time of firing is determined by the entire waveform of input current. **(C)** Sinusoidal current produces a smoothly accumulating change in the time-evolution of phase. A sine wave stimulus can advance or delay the next action potential, depending on the phase of the stimulus relative to each spike.

To determine whether differences in the PRCs could account for the differential entrainment of SP neurons and LTS interneurons, we constructed phase models from the individual PRCs of each neuron, and from the mean PRCs of the two cell types. This allowed us to simulate the entrainment and spiking resonance experiments using phase-model cells, and compare the simulation with experimental results from the same cells. The phase model is governed by a single differential equation,

$$\frac{d\,\varphi }{d\,t}=F_{C\,e\,l\,l}+\left(I_{S\,t\,i\,m}\,\left(t\right)+\eta \,\left(t\right)\right)\,Z\,\left(\varphi \right)$$

in which φ is the model cell’s phase, *F*_*Cell*_ is the cell’s unperturbed firing rate, *I*_Stim_(*t*) is the stimulus current, η(*t*) is the cell’s intrinsic membrane noise, and *Z*(φ) is the infinitesimal PRC measured experimentally. The evolution of phase in the model is illustrated in Figure 7 (bottom row). In the absence of any stimulus current, the cell’s phase evolves at a constant rate, *F*_*Cell*_. In this stimulus-free and noise-free configuration, phase and time co-evolve and the model fires rhythmically. Application of a brief current pulse produces a sudden shift in phase, the magnitude of which is determined by the amplitude and duration of the pulse and the value of the PRC at the phase of stimulus application, *Z*(φ). Because the stimulus alters the cell’s phase, later stimuli would arrive at phases that could not be predicted from their timing alone. Complex stimuli, like the sequence of random pulses shown in the center column, produce a correspondingly complex walk of phase through time, with each stimulus pulse altering the phase at which all subsequent stimulus pulses arrive. This disconnect between phase and time separates the phase model used here from those employing the weak-coupling approximation, in which the phase of any applied stimulus is assumed to be equal to its time of application normalized by the cell’s unperturbed period (e.g., Schwemmer and Lewis, 2011). All neurons have some intrinsic noise, caused by the stochastic opening of ion channels (White et al., 2000), and this limits the use of the weak-coupling approximation for predicting firing in real neurons, even for very simple inputs like single pulses (Ermentrout et al., 2011). The weak coupling approximation is more severely challenged by complex input waveforms like sinusoids, which cause a systematic deviation between phase and time, as shown in the right column of Figure 7. Accurate prediction of neuronal activity in response to these stimuli requires the more general version of the phase model used here, in which phase may evolve non-linearly in time. However, the general form is not amenable to closed-form mathematical solution and must be evaluated numerically.

### A Model of Entrainment

**FIGURE 8:**
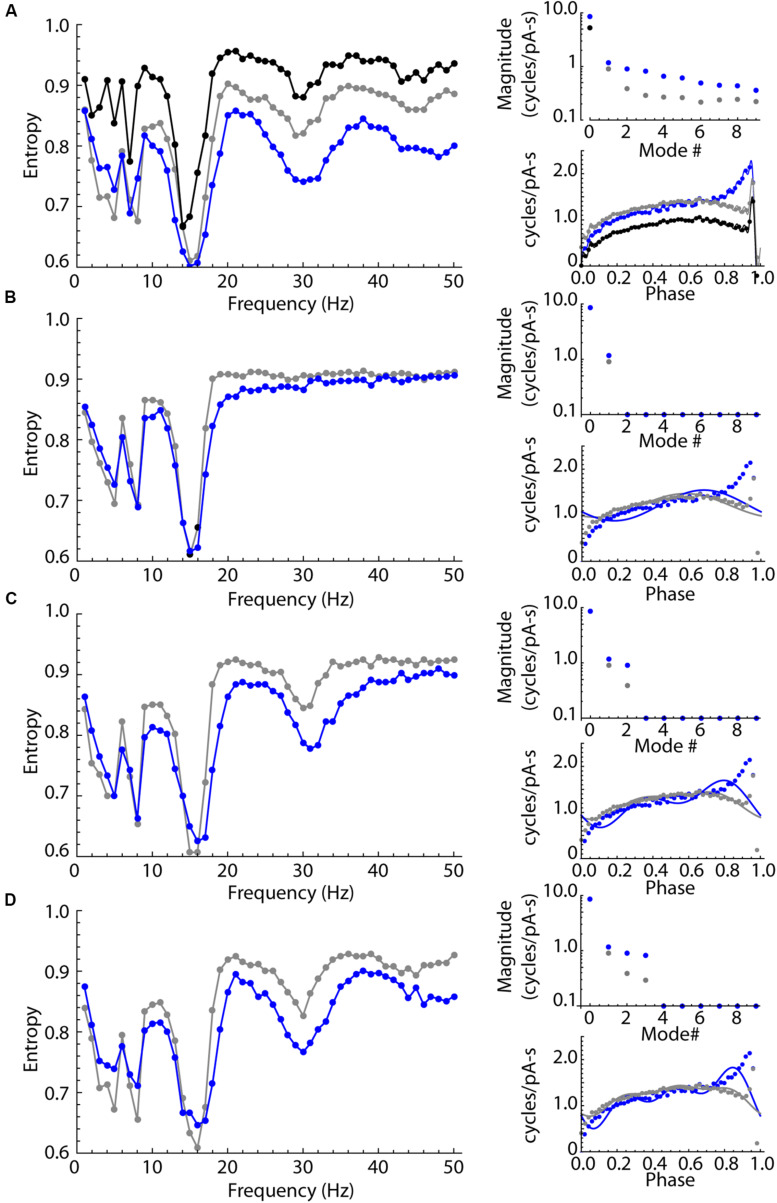
Sinusoidal entrainment in phase models of the LTS interneuron (in blue) and the SP neuron (in black and gray). **(A)** Entrainment spectra from 1 to 50 Hz for the phase model LTS interneuron (blue) and the SP neuron (black) using a phase model with all modes present. To test for the influence of the DC component mode 0, the SP neuron PRC was shifted to have the same mode 0 as the LTS interneuron, and the spectrum for that version of the SP neuron model is shown in gray. This mode 0 equalization brought the entrainment spectra mostly into alignment for frequencies at and below the cells’ unperturbed rate (15 spikes/s). The entrainment at higher frequencies continued to be different, with the SP neuron showing consistently higher entropy (less entrainment). The amplitudes of the first 10 modes in both PRCs are shown at right, including the shift in mode zero between the measured value for SP neurons (black), LTS neurons (blue) and the mode 0-equalized SP neuron (gray). The PRCs used in these simulations are shown at lower right in **(A)**. **(B)** The effect of equalizing mode 0 as above, and removing all modes higher than mode 1, which corresponds to the cell’s firing rate. At right are shown the amplitudes of modes and the synthetic PRCs (lines) superimposed on the original PRCs (points). The only difference between PRCs is the difference in amplitude and phase of mode 1. The cell type difference in entrainment at high frequencies is absent, except for a small range of frequencies near the cells’ firing rate. **(C)**. The same comparison except including differences in modes 1 and 2. The secondary (1:2) entrainment at twice the cells’ frequencies is returned and the difference in entrainment in that frequency range is restored. **(D)** Restoring modes 1 through 3 reproduces most of the original differences in high-frequency entrainment between the cell types.

The experimental results showed that LTS interneurons can be entrained by oscillatory inputs over a wider range of frequencies than SP neurons, but do not have a second frequency preference determined by their membrane impedance resonance. Possibly, this difference in entrainment arises from a difference in the shape or size of their PRCs. We used phase models based on the average PRCs of the SP neuron and LTS interneuron samples (the PRCs in Figures 6B,D) and repeated the experiments shown for real neurons in Figure 5 using these two neuron models. The unperturbed firing rate was set at 15 spikes/s in both cases, which was near the sample average for both cell types. The models included an estimate of the intrinsic noise of each cell type. On each time step of the integration, intrinsic noise was implemented by applying a current drawn from a normal distribution with a mean of zero. The standard deviation of the noise input was adjusted to reproduce the average coefficient of variation for our samples of SP neurons and LTS interneurons (0.10 and 0.12, respectively) in the absence of any stimulus using the method of Ermentrout et al. (2011). The results are shown in Figure 8A. Like the real neurons, the phase model of the SP neuron and the LTS neuron showed entrainment peaks at their unperturbed firing rates. They also showed smaller peaks near half and twice their firing rates, which were sometimes visible but less prominent in the real neurons. Also like the real neurons, the model LTS interneuron showed a much higher level of entrainment than SP neurons at all frequencies. As in real SP neurons, entrainment in the SP model was practically absent for input frequencies about 1.5 times the cell’s unperturbed rate. In this phase model, all differences in entrainment must be attributable to differences in the two cells’ PRCs.

Some of the difference in entrainment should result from the mean amplitude, rather than the shape of the PRC. The integral of the PRC (the amplitude of mode 0) is a measure of the response to the average current delivered during the interspike interval. It contributes to the response to sine waves of all periods except those that are integer multiples of the cell’s firing rate (for which the stimulus averaged over the interspike interval is zero). The overall larger PRC of the LTS cell (larger mode 0) is therefore expected to affect entrainment at nearly all stimulus frequencies. To determine the influence of mode 0, we shifted the SP neuron PRC by a constant value to equalize its average to that of the LTS interneuron (Figure 8A). Equalizing mode 0 reduced the difference in entrainment between LTS and SP models at all frequencies, and eliminated most of the differences between cell types at frequencies less than the cells’ firing rate. However, after equalizing mode 0, a substantial and consistent difference between LTS and SP models persisted at higher frequencies.

To dissect the contributions of PRC shape, we systematically eliminated frequency components of both cells’ PRCs. Figure 8B shows the result of eliminating all PRC modes except modes 0 and 1, while equalizing mode 0 as in Figure 8A so that the only difference remaining is in mode 1. Mode 1 is only slightly different in the two PRCs, and the remaining difference between the models was also small and restricted to frequencies near the cells’ firing rates. LTS interneuron and SP neuron PRCs differ greatly in the magnitude of mode 2. When each cell’s mode 2 was restored, the entrainment at twice the cells’ rates (about 30 Hz) returned, as did most of the difference in entrainment over the entire range above their firing rates. Additional replacement of mode 3 restored most of the remaining differences between models, especially at higher frequencies. Examination of the PRCs produced by reconstruction from modes 0–3 shows that these are not accurate reproductions of the original PRC shapes, but they do capture the large difference in the PRC skew between the cell types, including the LTS interneuron’s increase in the PRC at late phases. The similarity between the results from the phase model in Figure 8 and those from the real neurons in Figure 5 suggests that the entrainment of LTS interneurons may be fully explained by their phase resetting properties without reference to membrane impedance resonance.

### Spiking Resonance for Broadband Input in the Phase Model

**FIGURE 9:**
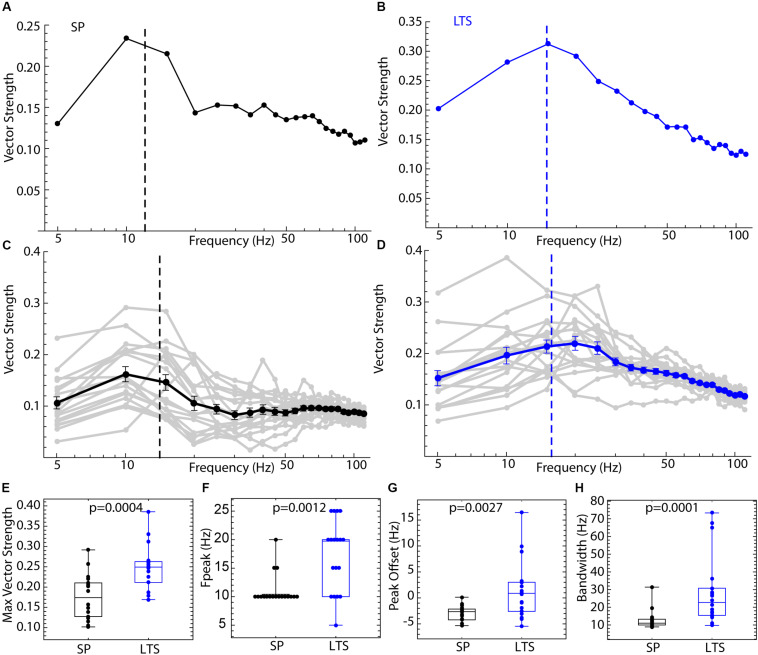
Spiking resonance for broadband input in the phase model. For each of the 18 LTS interneurons and 18 SP neurons, the experimental PRC and unperturbed firing rate were used to generate spike trains in response to pulsed noise stimuli like those used to measure spiking resonance in the real cells. **(A)** An example spiking resonance spectrum from a phase model based on an SP neuron. The cell’s firing rate is indicated by the dotted line. **(B)** An example phase model LTS interneuron shown the same way. **(C)** Superimposed spiking resonance spectra (gray) and the average spiking resonance (black) for 18 SP phase model neurons. **(D)** Superimposed individual spectra (gray) and average spectrum (blue) for phase models based on all 18 LTS interneurons. Error bars are standard errors of the mean at each frequency. The dotted lines indicate the average firing rates for the two groups of cells. **(E)** Model LTS interneurons showed a higher vector strength overall than model SP neurons. **(F)** The frequency at the peak resonance was higher in model LTS interneurons. **(G)** The difference between the firing rate and peak frequency for each cell was greater in model LTS interneurons. **(H)** The bandwidth of resonance (measured as shown in Figure 2) was greater in model LTS interneurons.

The difference in entrainment of LTS interneurons and SP neurons by sine wave input is attributable to differences in their PRCs, which could be reduced to the differences in the modes shown in Figures 6, 8. The differences between cell types in spiking resonance for broadband input share some resemblance to the frequency profile of entrainment by sine wave currents, as LTS interneurons showed much wider bandwidth of resonance and overall higher vector strength at all frequencies. To determine whether the differences in PRCs could explain these results, we repeated the spiking resonance experiment shown in Figure 3 using phase models generated for each of the cells. Current pulse noise was applied to phase models with firing rates and PRCs from the experimental sample, and spiking resonance spectra were calculated for each model cell. Examples are shown in Figures 9A,B, and the spectra for each cell and the mean spectrum are superimposed for each cell type in Figures 9C,D. The phase models reproduced all the differences between LTS interneurons and SP cells, including the differences in maximal amplitude, frequency at the peak, peak offset, and bandwidth, seen in the corresponding neurons as shown in Figures 9E–H. These results indicate that the cell type differences in frequency-selectivity were all attributable to differences in the size and shape of the PRCs.

## Discussion

The input to striatal neurons from the cortex and other areas contains oscillatory components, and cells in the striatum often fire in a consistent phase relationship with that input (Courtemanche et al., 2003; Kalenscher et al., 2010; Howe et al., 2011; Leventhal et al., 2012). Phase-locking depends on cell type, with fast-spiking interneurons usually locking to high or low parts of the gamma frequency range, and SP neurons usually related to lower frequencies (Berke et al., 2004; Sharott et al., 2009; van der Meer and Redish, 2009). The frequency selectivity of each interneuron type may be a key to its circuit function, at least in the feed-forward configuration in which interneurons and principal cells receive the same input. In that case, inhibition may reduce the response of principal cells to specific frequency components in the shared input, but not others.

The usefulness of frequency-based measurements is not restricted to understanding responses to periodic inputs. All realizable input waveforms, even noise, can be decomposed into sine wave components. If cells have a greater sensitivity to some frequencies than others, their responses to complex input waveforms are colored by that sensitivity. The frequency sensitivity of neurons is also a measure of how fast they respond to changes in the stimulus. Cells that can entrain to very high frequencies respond strongly and quickly to brief stimuli or abrupt changes in input.

### The Origin of Frequency Selectivity

Of course, the frequency selectivity of spike generation is not the only process imposing frequency selectivity on neurons in the striatal circuit. The frequency content of the input spike trains, synaptic current time course, dendritic filtering of synaptic input and frequency dependent short term synaptic plasticity all determine the frequency content of the current delivered to the spike generation mechanism. In our experiments, a broad range of frequencies were present and equally represented in the stimuli delivered to the soma, to isolate the frequency sensitivity of the spiking mechanism. In the real circuits, some frequencies will be represented more than others.

What determines the frequency selectivity of a neuron’s spiking response? Striatal SP cells have no subthreshold membrane resonance that could contribute to spiking resonance. Their spiking resonance and entrainment are the consequence of spike-triggered currents. Striatal LTS interneurons do have a membrane impedance resonance, but our results here show no influence of it on spiking resonance or entrainment when the cells are firing repetitively. Why does it not influence spiking? One possibility is that the membrane resonance of LTS neurons is engaged only at more depolarized membrane potentials. The calcium mechanism discovered by Song et al. (2016) to underlie membrane impedance resonance is engaged by ion channels that are not normally activated during the interspike interval. It is possible that membrane resonance could be influential in resumption of firing after a cell’s autonomous firing has been blocked in the persistent depolarized state (Song et al., 2016). This function would be consistent with known instances of spike patterning by membrane impedance resonance, in which firing is triggered on peaks of a noisy input applied to a cell near, but below the current threshold for repetitive firing (e.g., Kispersky et al., 2012; Linaro et al., 2018). In contrast, during repetitive firing, the responses of the non-resonant SP neuron and the resonant LTS interneuron could both be predicted using a phase-resetting model that includes no explicit subthreshold dynamics.

Of course, not all neurons *in vivo* are firing repetitively at any one time. In the basal ganglia there are several cell types that fire repetitively most or all of the time, because their firing is driven autonomously. Neurons in the output nuclei, the GPi and SNr, are all autonomously active, as are the neurons in the middle structures, the subthalamic nucleus and GPe (Surmeier et al., 2005; Wilson, 2015). In the striatum, the somatostatin positive LTS interneurons are autonomously active (Beatty et al., 2012), as are the cholinergic interneurons (Bennett and Wilson, 1999) and a class of burst-firing GABAergic interneurons (Assous et al., 2018). Other cells, including the SP neurons and fast-spiking interneurons, are not autonomously active but fire repetitively in brief episodes in response to stimuli or during movements. During those responses, firing rates transiently go well into the range of frequencies at which the cells fire repetitively (e.g., Kimura, 1990). When a neuron is firing repetitively, even for brief periods, it is possible to characterize input-driven changes in spiking using the phase resetting method (Wilson, 2017; Higgs and Wilson, 2019). The phase model of the neuron is not completely general and it is possible to force a repetitively firing neuron out of the range of its applicability (Kogh-Madsen et al., 2012). A host of experimental and environmental conditions might alter repetitive firing and the phase resetting process. The most obvious example would be an input that abolishes repetitive firing altogether. Neurons must be depolarized above rheobase to satisfy the assumptions of the phase model. However, it is not required that neurons fire rhythmically. A neuron that would fire rhythmically in the absence of perturbing stimuli but is densely perturbed by inputs and so fires irregularly may still be predicable by phase methods (Wilson et al., 2014).

In the phase resetting formulation, the unique properties of each neuron are encapsulated in its PRC. The results presented here show that differences in PRC shape can contribute to the characteristic frequency sensitivity differences between neurons. PRCs come in a variety of shapes, and there are a variety of ways to parameterize their shapes. Fourier components are not fundamentally better than any other method, but they are best suited for describing the responses to sinusoidal inputs or sinusoidal components of complex inputs (Goldberg et al., 2013). The results reported here show that the first few modes of the PRC are directly responsible for frequency sensitivity differences between LTS interneurons and SP neurons. For a neuron to respond to input frequencies higher than its own firing rate, its PRC must contain correspondingly high frequency components.

### Frequency-Selective Feed-Forward Inhibition

Striatal LTS interneurons provide feed-forward inhibition to the SP neurons, which are the striatal output cells. There is no recurrent excitation of interneurons by SP neurons, which are themselves GABAergic and inhibitory. SP neurons’ narrow frequency sensitivities are centered on their individual firing rates, and thus are easily tuned by rate (Wilson, 2017). At any one moment, there are SP neurons firing at a wide variety of rates, and so there are cells poised to respond differentially to a variety of frequency components in the synaptic input. SP neurons are also very numerous, so fragmenting the frequency sensitivities among the population of SP neurons by firing rate does not cause a great loss of representational capacity. LTS interneurons, on the other hand, are outnumbered by SP neurons by a factor of 100 or more (Tepper et al., 2010), and single LTS interneurons provide feed-forward inhibition to many SP neurons. The ability of LTS neurons to phase-lock to a wide range of frequencies may allow them to provide phase-locked inhibition to the much more numerous SP neurons a broad frequency range.

Compared to SP neurons, LTS interneurons are more sensitive to synaptic input, as indicated by the overall larger amplitude of their PRCs, and their firing is more phase-locked to periodic inputs at frequencies higher than their own firing rate as indicated by the higher magnitudes of their PRC modes above mode 1. The only exception to the LTS interneurons superiority is the SP neuron’s phase-locking to input frequencies near its own firing rate. The LTS interneuron is positioned to disrupt phase-locked firing in the SP neuron, by providing an inhibitory signal in phase with periodic excitatory inputs shared by both cell types. Because of its broadband response, the LTS neuron’s firing is most entrained by the largest-amplitude periodic signal in the input, and its inhibition of entrainment of SP neurons will be most effective for that frequency. This inhibition could further suppress the entrainment of SP neurons whose firing rates differ from the frequency of the dominant periodic input. Thus, the LTS interneuron could provide a broad surround inhibition to SP neurons, not in space, but in the frequency domain.

## Data Availability Statement

The datasets generated for this study are available on request to the corresponding author.

## Ethics Statement

The animal study was reviewed and approved by Institutional Animal Care and Use Committee of the University of Texas at San Antonio.

## Author Contributions

JM, MH, SS, and CW designed the experiments and formulated the analysis. JM, SS, and CW collected the data and performed the analysis. JM, MH, SS, and CW wrote the manuscript.

## Conflict of Interest

The authors declare that the research was conducted in the absence of any commercial or financial relationships that could be construed as a potential conflict of interest.
